# Chloroform—Preventive of Puerperal Mania

**Published:** 1853-04

**Authors:** 


CHLOROFORM—PREVENTIVE OF PUERPERAL MANIA.

Dr. Simpson believes, that to the many other valuable properties of chloroform may be added the power of preventing puerperal mania. He has lately communicated to the Obstetrical Society of Edinburgh three observations relating to women, who, having previously suffered with this malady after confinement, were delivered under the influence of chloroform without any such unpleasant consequence. One of these women, the mother of a large family, had never escaped an attack of the disease in her former confinements.

